# Study on molecular mechanism of ANOS1 promoting development of colorectal cancer

**DOI:** 10.1371/journal.pone.0182964

**Published:** 2017-08-30

**Authors:** Lu Qi, Wenjuan Zhang, Zhiqiang Cheng, Na Tang, Yanqing Ding

**Affiliations:** 1 Department of Pathology, School of Basic Medical Sciences, Southern Medical University, Guangzhou, China; 2 Department of Pathology, Shenzhen People's Hospital, Shenzhen, China; 3 Department of Pathology, Nanfang Hospital, Southern Medical University, Guangzhou, China; National Cancer Center, JAPAN

## Abstract

Our main aim was to elucidate the molecular mechanism responsible for the development and metastasis of colorectal cancer. Furthermore, we also identified the key genes participating in this molecular mechanism and stimulating the progression of colorectal cancer. In our experiment, the ANOS1 gene showed up-regulated expression levels continuously, whereas the methylation level showed downregulated levels when the colorectal cancer progressed through the four clinical stages of development and metastasis. We obtained this information by analyzing the expression profile data and methylation data of ANOS1 gene in colorectal cancer. This phenomenon indicates that ANOS1 gene shows continuous activation during the progression of colorectal cancer. According to the results of survival analysis, the expression of ANOS1 gene is closely related to the overall survival rate of patients (p = 0.003); moreover, the expression of ANOS1 gene is also strongly associated with the disease-specific survival rate (p = 0.001). When the expression of ANOS1 gene is high, the survival rate is low in patients. When the expression of ANOS1 gene is low, the survival rate of patients is high. To elucidate the possible molecular mechanism of ANOS1, we performed GSEA enrichment analysis based on the expression value of ANOS1 gene. We found that gene with an up-regulated expression was mainly involved in Wnt signaling pathway; these up-regulated genes were present in the group of high ANOS1 expression. Although several genes were involved in Wnt signaling pathway, CTHRC1 gene was of higher occurrence frequency. By co-expression analysis, we found that both expression value and expression extent of CTHRC1 were associated with ANOS1. All these results indicate that ANOS1 possibly promotes the activation of Wnt signaling pathway with its co-expression partner CTHRC1. Thus, ANOS1 and CTHRC1 genes promote the development and metastasis of colorectal cancer.

## Introduction

The gene ANOS1 is also known as KAL1, and it is located in X chromosome. Encoded proteins are located in extracellular matrix; the encoded protein sequences are similar to proteins with nerve cell adhesion and axon migration function. Previous studies have reported that this protein is closely associated with cell adhesion. In few reports, it was proved that ANOS1 was related to tumor. For example, some studies reported that the expression of ANOS1 is up-regulated in gastric cancer tissue. By inhibiting the expression of ANOS1, scientists could reduce the proliferation, invasion, and migration ability of gastric carcinoma cell[1]. However, some studies have reported that the development of oral squamous cell carcinoma is promoted with a low expression of ANOS1 gene.[2] The ANOS1 protein is located in extracellular matrix, and it plays a pivotal role in cell adhesion and migration. In totality, previous studies have reported that ANOS1 possibly plays an important role in the development and metastasis of cancer. Colorectal cancer is one of the most common malignant tumors in the digestive tract of humans. In addition, metastasis is the most important factor for the death of patients with colorectal cancer. Because ANOS1 expression is rarely investigated in patients with colorectal cancer, we decided to focus our attention on ANOS1 in this study. We determined the expression and methylation levels of ANOS1 gene by compiling the expression profile data and methylation data of patients with colorectal cancer. Furthermore, we investigated the relationship between ANOS1 gene expression and patient’s survival time. For this purpose, we performed survival analysis and GSEA[3, 4] enrichment analysis. Thus, we elucidated the possible molecular mechanism through which ANOS1 instigates the progression of colorectal cancer in patients.

## Materials and methods

### Change in expression level and methylation level of ANOS1 in colorectal cancer

A gene expression level difference exists between different the various stages of colorectal cancer progression. To identify the different changes that occur in the expression level and methylation level of ANOS1 gene during the various stages of colorectal cancer progression, we extracted the gene expression data of ANOS1 from the expression profile data [GSE41258] of colorectal cancer in Gene Expression Omnibus (GEO)[5] database (S1 Table). In addition, we also extracted the methylation data of ANOS1 gene from the methylation level data of colorectal cancer, which was present in the Cancer Genome Atlas (TCGA)[6] database (S2 Table). Interestingly, GSE41258 contained the data of 54 normal colorectal cases, 28 cases of stage I colorectal cancers, 50 cases of stage II colorectal cancers, 49 cases of stage III colorectal cancers, and 58 cases of stage IV colorectal cancers. Methylation data contained the data of 32 cases of stage I colorectal cancers, 64 cases of stage II colorectal cancers, 45 cases of stage III colorectal cancers, and 24 cases of stage IV colorectal cancers. The one-way analysis of variance (ANOVA) was conducted to determine the gene expression and methylation data of ANOS1 in the above two groups, respectively. Thereafter, we plotted the data of the two groups and analyzed the results to arrive at a conclusion.

### Influence of ANOS1 on the survival of patients with colorectal cancer

To decipher the influence of ANOS1 expression level on the survival of patients with colorectal cancer, we conducted a survival analysis for ANOS1 gene by compiling an expression profile data GSE17536 in GEO database of this study. The GSE17536 data package contained information about 177 cases with respect to the following parameters: i) overall survival data and ii) disease-specific survival data. The various values of ANOS1 gene expression were extracted from the expression profile data (S3 Table). Then, they were uniformly divided into two groups: a high expression group and a low expression group based on the median after sorting. There were 88 cases of data in each group. To obtain the survival curve, we analyzed the survival difference between the two groups using Kaplan-Meier[7, 8] survival analysis method. The analysis software SPSS 17.0 was used in this experiment.

### Analysis of molecular mechanism of ANOS1 in colorectal cancer

To elucidate the underlying molecular mechanism through which ANOS1 stimulates the progression of colorectal cancer, we analyzed the molecular mechanism of ANOS1 using the expression profile data GSE39582 in GEO database. The GSE39582 date package included the expression profile data of 566 cases of colorectal cancer tissues. To elucidate the influence of ANOS1 gene expression on the colorectal cancer expression profile, the expression profile data of 566 cases were sorted in terms of ANOS1 gene expression value. Then, they were uniformly divided into two groups: a high expression group and a low expression group. Each group contained data of 283 cases. An enrichment analysis was conducted in the biological process of gene ontology using GSEA tool for the two groups of data; the gene set version was V5.2.

## Results

### Changes in expression and methylation level of ANOS1 in colorectal cancer

**Fig 1:**
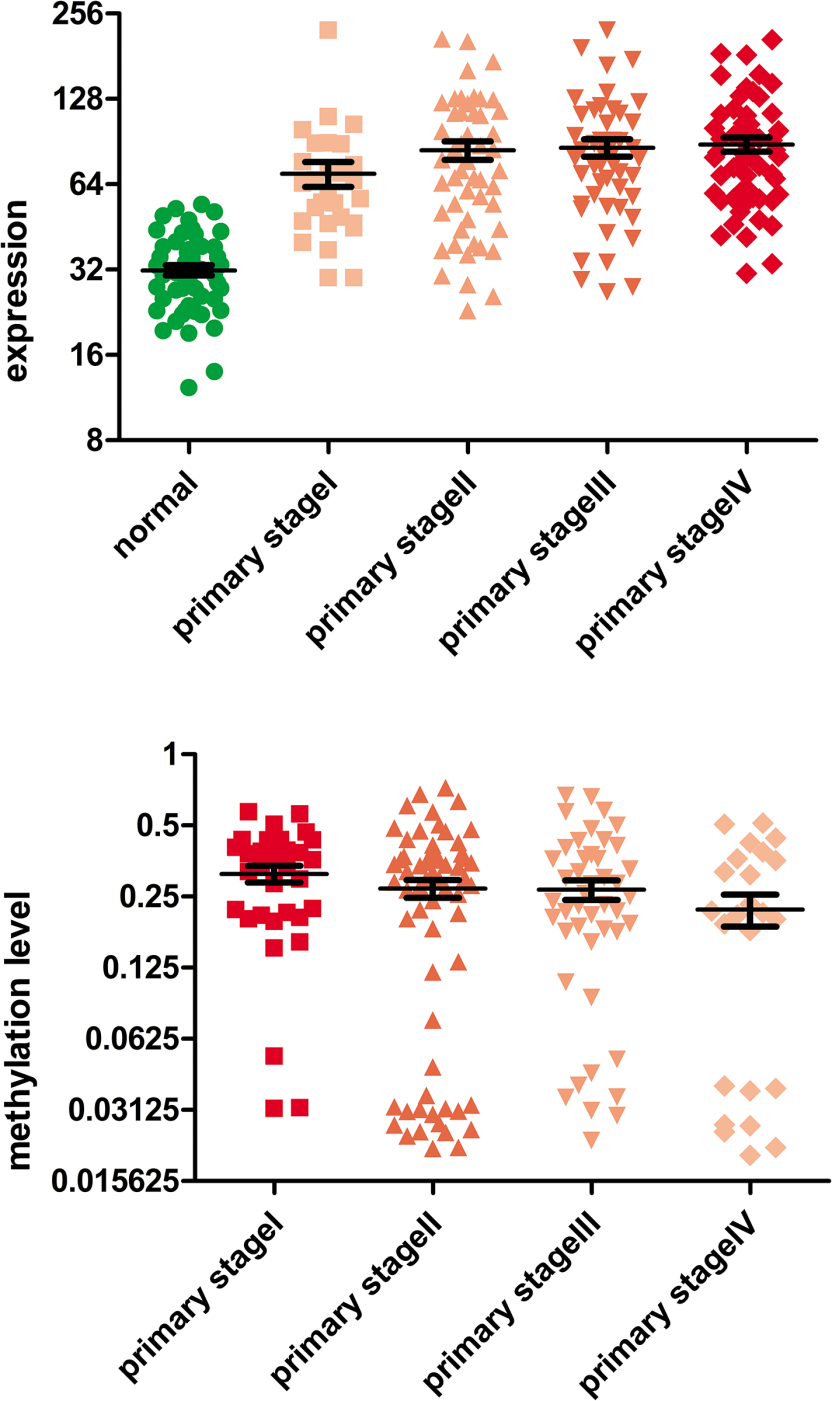
The continuous increase in the expression level of ANOS1 gene during the progression of colorectal cancer; however, the methylation level continues to decrease as colorectal cancer progresses in patients.

We found that there was a significant difference between the expression level and methylation level of ANOS1 gene in normal tissue and different stages of colorectal cancer (P < 0.0001). With the progression of colorectal cancer, the expression level of ANOS1 gene continued to increase but the methylation level continued to decrease (Fig 1). An increase in the gene expression level indicated gene activation. Moreover, a decrease in methylation level also indicated gene activation. Since both the expression level and methylation level of ANOS1 gene underwent dramatic changes, we infered that ANOS1 gene was in a state of continuous activation during the progression of colorectal cancer. This indicates that ANOS1 gene played a very important role in colorectal cancer.

### Influence of ANOS1 on the survival of patients with colorectal cancer

**Fig 2:**
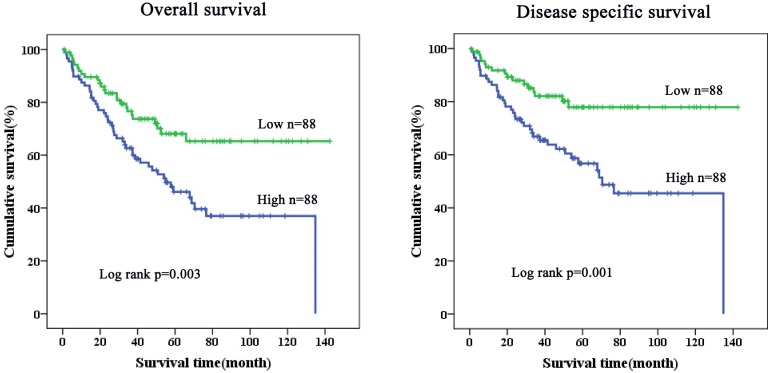
The influence of ANOS1 gene on the survival of patients with colorectal cancer.

From the survival analysis, we found that gene expression of ANOS1 was related to the overall survival rate (p = 0.003), and it was also associated with disease-specific survival rate (p = 0.001). When the expression of ANOS1 gene was high, the patients’ survival rate was low. On the other hand, when the expression of ANOS1 gene was low, the patients’ survival rate was high (Fig 2). This indicates that that the expression level of ANOS1 gene was closely related to the progression of colorectal cancer. Moroever, it also indicates that ANOS1 gene undergoes continuous activation, leading to the promotion of tumors that cause colorectal cancer. Thus, ANOS1 gene plays an important role in the development of colorectal cancer.

### Molecular mechanism of ANOS1 in colorectal cancer

**Fig 3:**
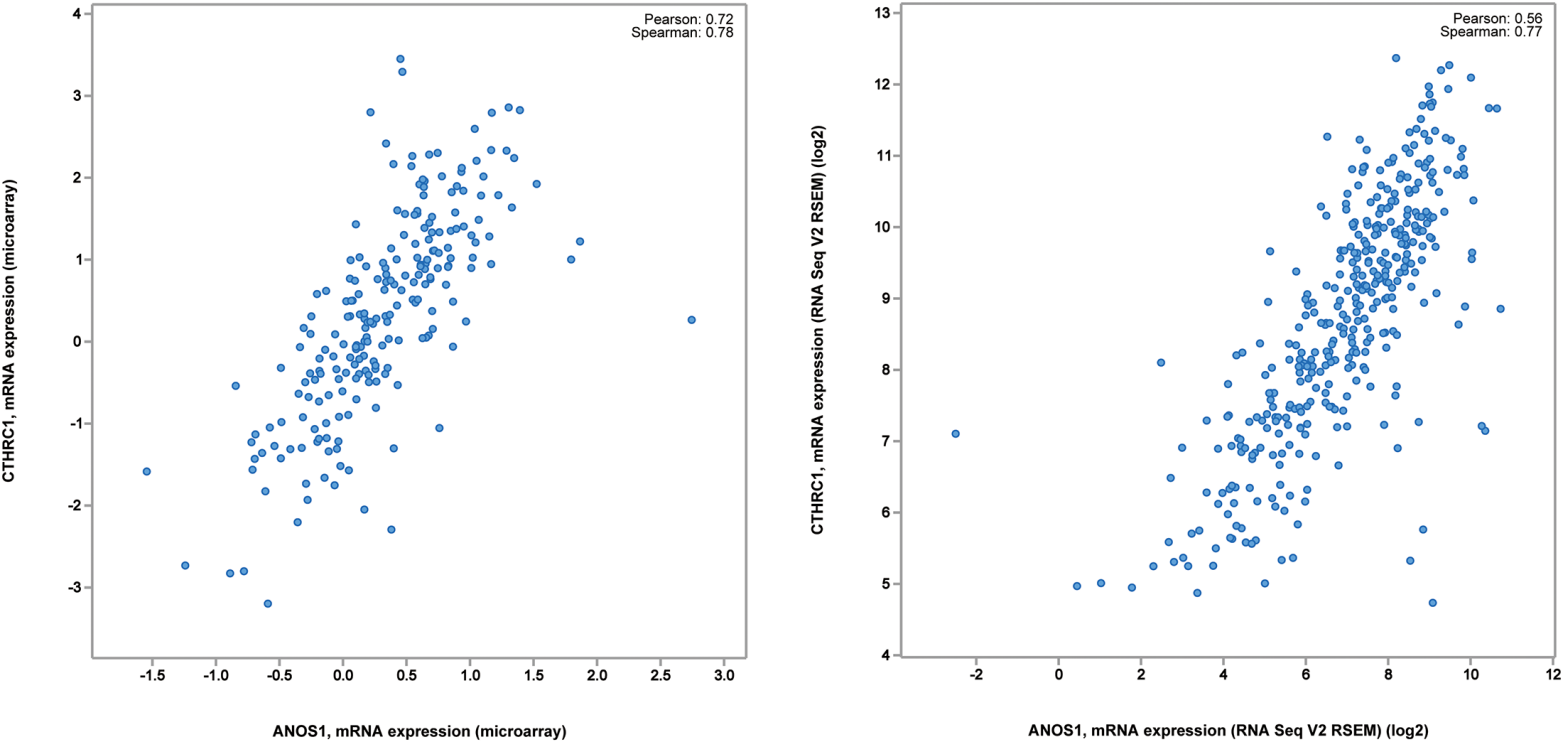
The figure demonstrated the correlation between ANOS1 and CTHRC1. And the relatively high correlation between them was revealed both in the gene chip data and RNAseq data.

**Fig 4:**
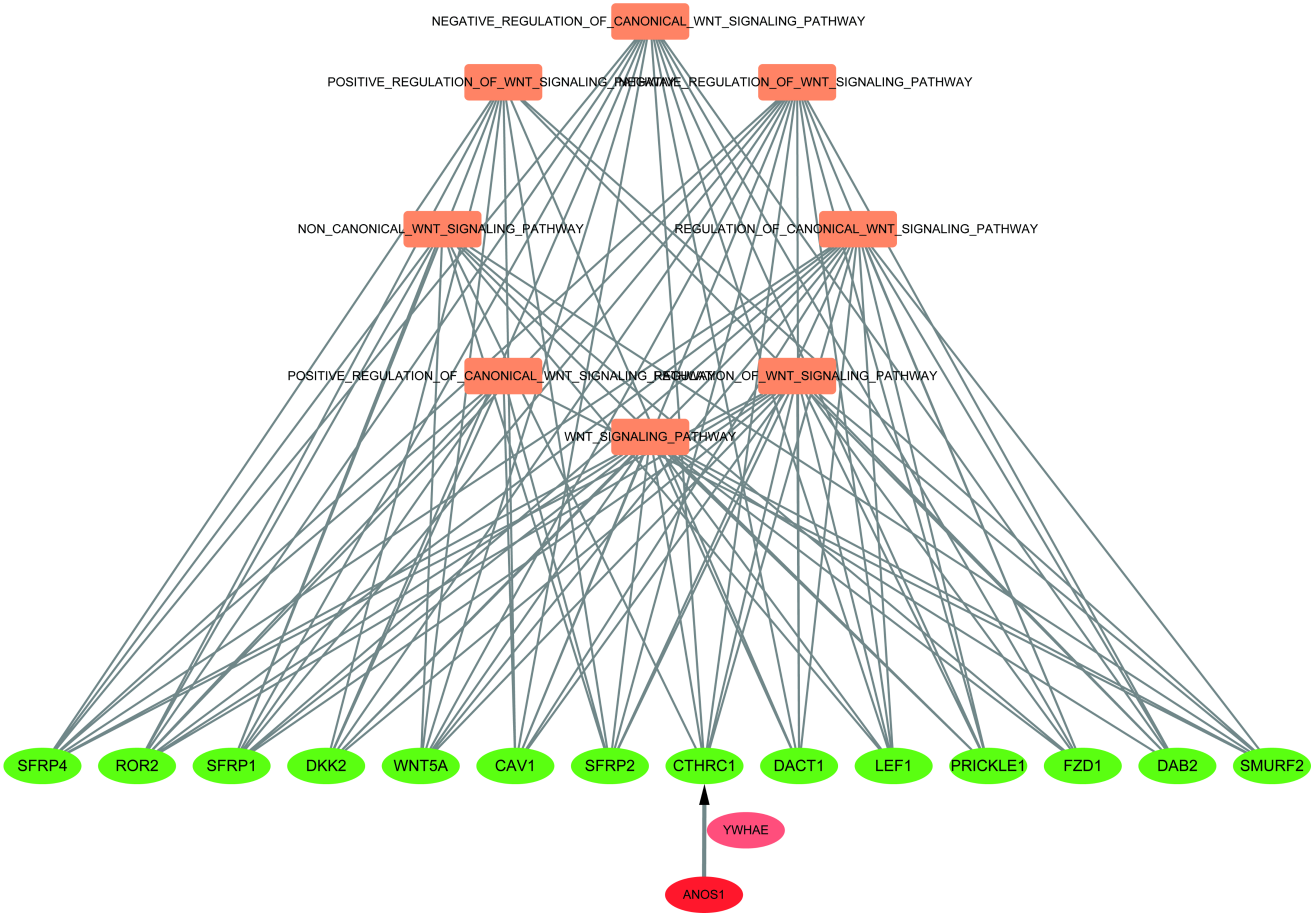
The underlying molecular mechanism through which ANOS1 participates in the regulation of Wnt signaling pathway.

In this experiment, GSEA enrichment analysis was used to evaluate the difference in the expression level of ANOS1 gene; while analyzing the biologically enriched classes, we found that most up-regulated gene sets were concentrated in the high expression group of ANOS1 gene. In the first 12 highest-score enrichment results, we detected eight Wnt signaling pathways relevant to biological processes (Table 1). Among the eight Wnt signaling pathway biological processes, the following 14 genes had an occurrence number greater than 6: SMURF2, SFRP2, DAB2, SFRP1, CAV1, ROR2, WNT5A, FZD1, PRICKLE1, LEF1, DACT1, SFRP4, CTHRC1, and DKK2. Furthermore, the co-expression partners of ANOS1 gene were screened using cBioPortal[9] tool: we analyzed the data of 633 cases of colorectal cancer in TCGA database. After combining the microarray data and RNA-sequencing data, we found that the the co-expression of CTHRC1 and ANOS1 genes was the highest among all the 14 genes mentioned earlier. According to the microarray data, the Pearson coefficient of CTHRC1 and ANOS1 co-expression was 0.72 and the Spearman coefficient was 0.78. Furthermore, RNA-seq sequencing data showed that Pearson coefficient of CTHRC1 and ANOS1 co-expression was 0.56 and Spearman coefficient was 0.77. This indicates that correlations existed between CTHRC1 and ANOS1 in terms of value and changing trend of expression levels (Fig 3). The above results indicates that the molecular mechanism of ANOS1 is associated with the progression of colorectal cancer, and it most probably proceeded through the Wnt signaling pathway. Furthermore, the results also suggest that ANOS1 has a higher correlation with CTHRC1 in Wnt signaling pathway (Fig 4).

**Table 1:** The enrichment of biological processes in the high expression group of ANOS1 gene.

NAME	SIZE	ES	NES	NOM p-val	FDR q-val	FWER p-val
GO_REGULATION_OF_ESTABLISHMENT_OF_PLANAR_POLARITY	108	0.690345	2.25244	0	0.019824	0.009
GO_POSITIVE_REGULATION_OF_CANONICAL_**WNT_SIGNALING_PATHWAY**	106	0.613516	2.226777	0	0.013731	0.01
GO_POSITIVE_REGULATION_OF_**WNT_SIGNALING_PATHWAY**	136	0.623127	2.225323	0	0.009154	0.01
GO_NEGATIVE_REGULATION_OF_CANONICAL_**WNT_SIGNALING_PATHWAY**	155	0.669632	2.175398	0	0.017115	0.018
GO_REGULATION_OF_CANONICAL_**WNT_SIGNALING_PATHWAY**	214	0.636237	2.153054	0	0.015836	0.021
GO_**WNT_SIGNALING_PATHWAY**	332	0.561135	2.152725	0	0.013197	0.021
GO_NON_CANONICAL_**WNT_SIGNALING_PATHWAY**	138	0.593559	2.14884	0	0.012139	0.022
GO_REGULATION_OF_ORGAN_MORPHOGENESIS	232	0.661194	2.137563	0	0.013684	0.024
GO_REGULATION_OF_**WNT_SIGNALING_PATHWAY**	277	0.617697	2.132787	0	0.013437	0.025
GO_CELL_JUNCTION_ORGANIZATION	170	0.584207	2.101897	0	0.01485	0.033
GO_ESTABLISHMENT_OF_PROTEIN_LOCALIZATION_TO_MEMBRANE	238	0.371482	2.082741	0	0.018065	0.047
GO_NEGATIVE_REGULATION_OF_**WNT_SIGNALING_PATHWAY**	185	0.63165	2.079139	0	0.01689	0.047

**Fig 5:**
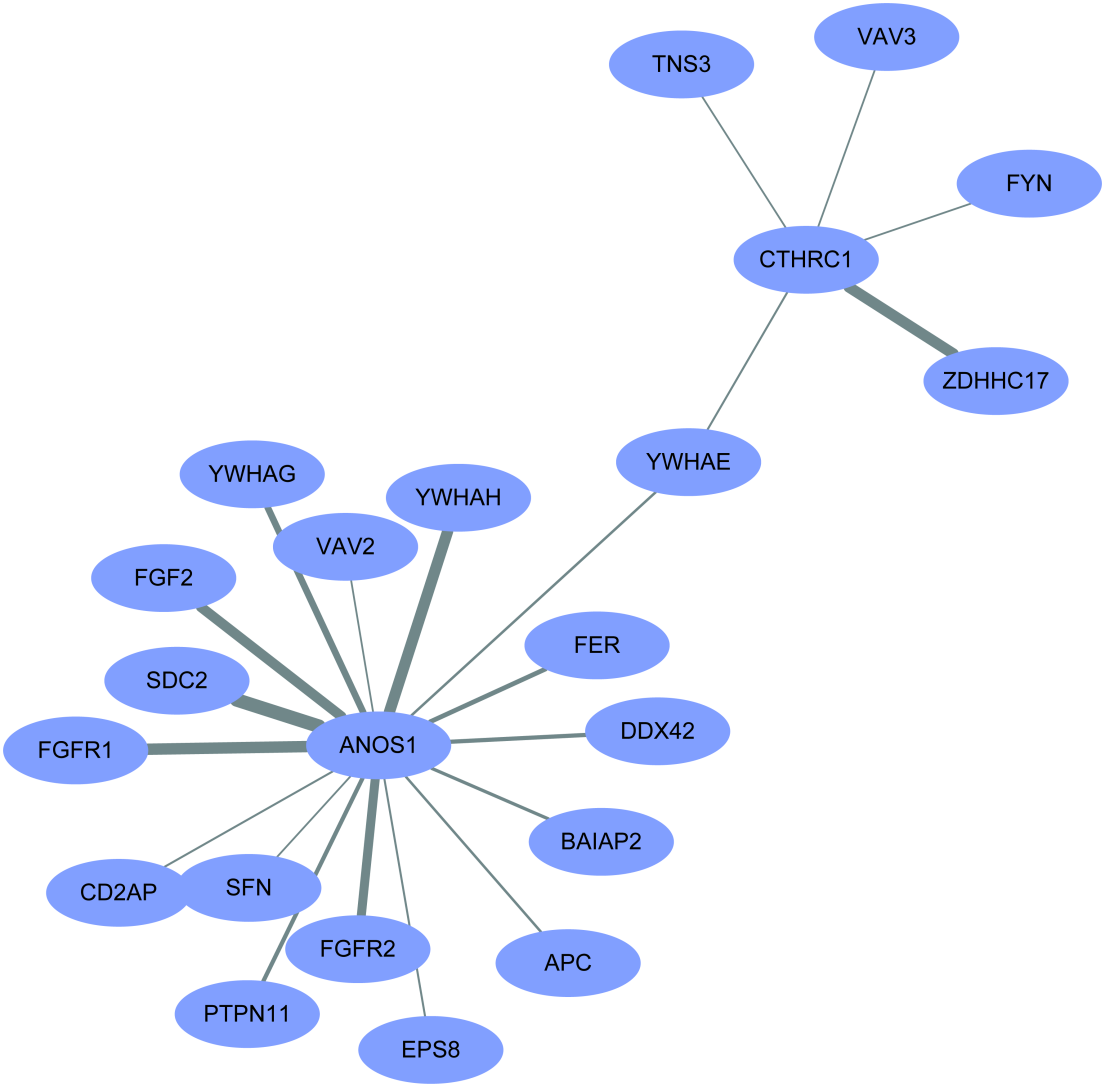
As shown in the figure for the protein interaction between ANOS1 and CTHRC1, they might interact with each other via YWHAE.

Because only a few proteins interact with CTHRC1 and ANOS1, the proteins interacting with ANOS1 and CTHRC1 were predicted using PrePPI tool. The predicted results showed that The target protein YWHAE was the only protein that was common to both ANOS1 and CTHRC1 as it showed interactions with both the genes. Therefore, ANOS1 was linked to CTHRC1 *via* YWHAE (Fig 5).

## Discussion

Very few studies have investigated the role of ANOS1 gene in the development of tumors. Moreover, only a handful of studies might have investigated the relationship between colorectal cancer and the expression of ANOS1 gene. In this study, we found that ANOS1 exhibits continuous elevation in its expression levels. Meanwhile, methylation levels decrease continously. This observation on ANOS1 gene and methylation levels was done while investigating the four tumor stages associated with colorectal cancer. The results indicate that ANOS1 gene shows continous activation when there is progression of colorectal cancer in patients. While performing survival analysis, we found that the survival rate of a patient is lower when the expression of ANOS1 is higher. On the other hand, the survival rate of a patient is higher when the expression of ANOS1 is lower. Based on these observations, we conclude that ANOS1 plays an extraordinary role in the progression of colorectal cancer. Since ANOS1 is located in the extracellular matrix, it may be probably involved in the tumor microenvironment constitution. Moroever, ANOS1 gene plays an important role in cell adhesion, cell migration, and molecular events that are closely related to tumor metastasis. After performing GSEA analysis on the expression profile data of colorectal cancer cases, we divided the results into two classes based on the expression value of ANOS1 gene. In the high expression group of ANOS1 gene, the genes with an up-regulated expression participate in biological processes. These genes are mainly involved in the processes through the Wnt signaling pathway. The occurence frequency of CTHRC1 gene is higher in multiple Wnt signaling pathway associated with enrichment classes. Furthemore, CTHRC1 gene is positively correlated with ANOS1. This implies that CTHRC1 probably plays an important role in the gene function/process of ANOS1. Many studies have investigated the role of CTHRC1 in colorectal cancer. These studies have reported that the proliferation, invasion, and migration of colorectal cancer cells is stimulated with an overexpression of CTHRC1 gene[10, 11]. In addition, the survival rate of patients is lower when they show a high expression of CTHRC1[12]. All these results indicate that CTHRC1 plays a pivotal role in promoting the progression of colorectal cancer. In this study, we found that the correlation between the expression of ANOS1 and CTHRC1 genes is higher among the 14 genes mentioned earlier. Moreover, ANOS1 and CTHRC1 have a common target protein YWHAE, which is confirmed by prediction results. All these results confirm that there is a strong correlation between ANOS1 and CTHRC1. Many genes show changes in expression levels during the progression of colorectal cancer; however, few genes exhibit a continuous up-regulation in their expression levels during the development of tumor. Furthermore, few genes exhibit continuous down-regulation in methylation during the development of tumor. Furthermore, ANOS1 gene not only meets such requirements, but it is also closely related to the survival time of patients with colorectal cancer. Moreover, it is assocaited with the classic signaling pathway (Wnt signaling pathway) of tumor. All these evidences prove that ANOS1 gene is a very important factor that needs to be further investigated in future research studies of colorectal cancer. We need to carry out comprehensive research studies on this gene in order to decipher the molecular mechanism responsible for the development and metastasis of colorectal cancer. Finally, we would like to reiterate that ANOS1 gene can be a guiding factor in the clinical treatment of colorectal cancer.

## Supporting information

S1 Table ANOS1 expression data were extracted from GEO database GSE41258. (XLSX)

S2 Table ANOS1 methylation βvalue were extracted from TCGA. (XLSX)

S3 Table ANOS1 survival data were extracted from GEO database GSE17536. (XLSX)
